# Identification and molecular characterization of *Mycobacterium bovis* DNA in GeneXpert® MTB/RIF ultra-positive, culture-negative sputum from a rural community in South Africa

**DOI:** 10.1016/j.onehlt.2024.100702

**Published:** 2024-03-03

**Authors:** Wynand J. Goosen, Sashen Moodley, Giovanni Ghielmetti, Yumna Moosa, Thando Zulu, Theresa Smit, Vukuzazi Team, Leanie Kleynhans, Tanya J. Kerr, Elizabeth M. Streicher, Willem A. Hanekom, Robin M. Warren, Emily B. Wong, Michele A. Miller

**Affiliations:** a Department of Science and Innovation – National Research Foundation Centre of Excellence for Biomedical Tuberculosis Research, South African Medical Research Council Centre for Tuberculosis Research, Division of Molecular Biology and Human Genetics, Faculty of Medicine and Health Sciences, Stellenbosch University, PO Box 241, Cape Town 8000, South Africa; b Africa Health Research Institute, KwaZulu-Natal, South Africa; c Section of Veterinary Bacteriology, Institute for Food Safety and Hygiene, Vetsuisse Faculty, University of Zurich, Winterthurerstrasse 270, 8057 Zurich, Switzerland; d Mater Research Institute - The University of Queensland, Translational Research Institute, Brisbane, QLD 4102, Australia; e Division of Infection and Immunity, University College London, London, UK; f Division of Infectious Diseases, Department of Medicine, Heersink School of Medicine, University of Alabama Birmingham, Birmingham, AL, USA

**Keywords:** Hluhluwe-iMfolozi park, *Mycobacterium bovis*, Spoligotyping, Tuberculosis control, Wildlife-livestock-human interface, Zoonotic transmission

## Abstract

This study investigated the presence of *Mycobacterium bovis* (*M. bovis*) DNA in archived human sputum samples previously collected from residents who reside adjacent to the *M. bovis*-endemic Hluhluwe-iMfolozi wildlife park, South Africa (SA). Sixty-eight sputum samples were GeneXpert MTB/RIF Ultra-positive for *M. tuberculosis* complex (MTBC) DNA but culture negative for *M. tuberculosis*. Amplification and Sanger sequencing of *hsp65* and *rpoB* genes from DNA extracted from stored heat-inactivated sputum samples confirmed the presence of detectable amounts of MTBC from 20 out of the 68 sputum samples. Region of difference PCR, spoligotyping and *gyrB* long-read amplicon deep sequencing identified *M. bovis* (*n* = 10) and *M. tuberculosis* (*n* = 7). Notably, *M. bovis* spoligotypes *SB0130* and *SB1474* were identified in 4 samples, with *SB0130* previously identified in local cattle and wildlife and *SB1474* exclusively in African buffaloes in the adjacent park. *M. bovis* DNA in sputum, from people living near the park, underscores zoonotic transmission potential in SA. Identification of spoligotypes specifically associated with wildlife only and spoligotypes found in livestock as well as wildlife, highlights the complexity of TB epidemiology at wildlife-livestock-human interfaces. These findings support the need for integrated surveillance and control strategies to curb potential spillover and for the consideration of human *M. bovis* infection in SA patients with positive Ultra results.

## Highlights

• First evidence of *Mycobacterium bovis* DNA in South African human sputum near wildlife parks.
• Zoonotic transmission potential in TB-endemic communities adjacent to wildlife.
• *M. bovis* spoligotypes *SB0130* and *SB1474* highlights transmission complexity.
• Integrated surveillance crucial in high-risk TB regions.
• *M. bovis* DNA in TB-positive Ultra results in South Africa.

## 1 Introduction

*Mycobacterium bovis* (*M. bovis*), a versatile zoonotic pathogen primarily associated with cattle, is responsible for bovine tuberculosis (bTB) [1]. However, its host range extends far beyond cattle, encompassing other domesticated animals, wildlife species, and even humans [2,3]. While high-income nations have made significant strides in eradicating the disease, it continues to proliferate in lower and middle-income countries. This persistence strains economies and jeopardizes livelihoods [4]. Maintenance of *M. bovis* in wildlife further complicates control efforts, particularly at interfaces where wildlife, livestock, and humans converge [5]. These interfaces emerge as a consequence of human activities encroaching into wildlife conservation zones or moving closer to the borders of *M. bovis*-endemic wildlife parks, increasing the likelihood of human-livestock-wildlife interactions [6].

Cross-species transmission of *M. bovis* has become a documented reality at these interfaces, illustrated by instances of transmission between African buffaloes (*Syncerus caffer*), other wildlife species, and domestic livestock in South Africa (SA) [6]. Even in first world countries, diverse wildlife species have emerged as substantial sources of infection, challenging efforts to control and eliminate bTB in cattle [7].

The transmission of *M. bovis* can occur through the inhalation of contaminated aerosols, or the consumption of contaminated food and water sources [6,8]. The African buffalo population in Hluhluwe iMfolozi Park (HiP) serves as a reservoir for *M. bovis*, leading to spill-over infections in various wildlife species [2]. Genetic analysis of *M. bovis* isolates from HiP's buffalo population has unveiled buffalo specific (SB*1474*) and shared (SB*0130*) spoligotype patterns with communal cattle. The latter indicating cross-species transmission at the wildlife-livestock interface [6,9]. In SA, the absence of an effective bTB control program in communal cattle, coupled with the consumption of contaminated raw animal products (both livestock and wildlife) and a high prevalence of HIV/AIDS among the population (prevalence estimates of 34.2%), escalates the risk of zoonotic TB in communities residing at these interfaces [10]. It is important to note that most international TB surveillance programs, including those in SA's rural communities, often do not employ culture medium or incubation times optimal for the growth of *M. bovis* and may not routinely collect extra-pulmonary samples for evaluation [11,12]. Remarkably, to date, in SA there have been no documented reports of *M. bovis* in people [13]. This observation is indeed perplexing, especially when considering increased reports of *M. bovis* infections in people from at-risk communities in neighbouring African countries [14,15], the high prevalence of HIV/AIDS among SA populations, the well-established presence of *M. bovis* in SA wildlife and livestock, and the recent surge in environmental reports documenting its presence in wastewater [16].

Genotyping techniques play a pivotal role in epidemiological investigations, enabling the tracing of infection sources, the delineation of pathogen circulation in specific populations, and the mapping of transmission routes [17]. In recent years, the integration of highly sensitive PCR methods, including the GeneXpert MTB/RIF Ultra qPCR assay (Ultra), alongside refined *Mycobacteria* spp. genus-specific PCRs, Region of Difference PCRs (RD-PCRs), *gyrB* PCRs, and spoligotyping, has represented a significant advance in molecular epidemiological research, spanning both human subjects and animals [9,[18], [19], [20], [21]]. Notably, the incorporation of IS*1081* as an additional target, alongside IS*6110* in the Ultra assay, has markedly broadened its applicability, aiding in the identification of *M. bovis* in diverse sample types obtained from both humans and various animal species [20,22,23,24,25].

Our study occurred within the Hlabisa sub-district in the Umkhanyakude District Municipality of the KwaZulu-Natal province (SA), a region of high HIV endemicity [26,27]. This area is characterized by vast communal farmland intricately interwoven with a public game reserve known as HiP, where *M. bovis* infections in numerous wildlife species have been well-documented over the years [6,8]. The HiP encompasses a publicly used corridor road (R618) that traverses the park, and is responsible for numerous wildlife incursions into neighbouring communities [28]. Recently, the significance of these incursions has been underscored by the detection of shared *M. bovis* strains between wildlife and traditionally farmed cattle within this wildlife-livestock interface [6,9]. Unlike other African countries, there is limited information on interspecies *M. bovis* transmission between wildlife, livestock, and humans in such at-risk neighbouring communities in SA [4,8,29].

Therefore, this pilot study aimed to detect the presence of *M. bovis* DNA from archived heat-inactivated sputum specimens and, upon confirmation, attempt to identify which *M. bovis* specific strains were potentially circulating among people residing in the Hlabisa sub-district.

## 2 Materials and methods

### 2.1 Study area and patient subgroup

The study area is the Africa Health Research Institute Demographic Surveillance Area (AHRI DSA) in the Hlabisa sub-district in Umkhanyakude District Municipality, KwaZulu-Natal province of South Africa, one of the world's most intensely HIV-affected regions (prevalence of 34.2%) [27]. The area is defined as a wildlife-livestock-human interface due to communal subsistence farmland bordering the well-known *M. bovis* endemic HiP [6]. For this pilot study, archived, decontaminated and heat-inactivated sputum pellets were selected from an existing biorepository generated during a multi-disease population-based screening study called Vukuzazi between 2018 and 2020 [27]. In this study, a comprehensive medical history was collected, spanning conditions such as general health status, TB history, TB symptoms, X-ray findings, smoking status, alcohol consumption and HIV status [27]. All participants provided written informed consent [27].

### 2.2 Sputum processing, mycobacterial culture, ultra testing, and DNA extraction

**Fig. 1:**
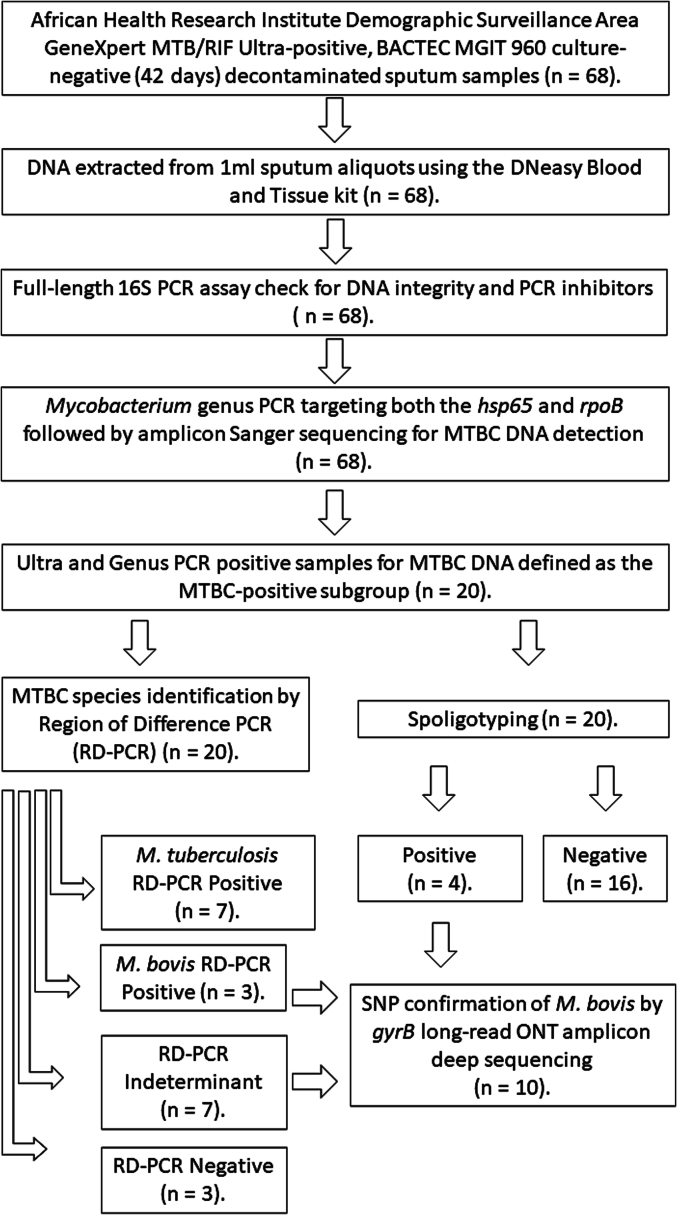
Study method flow chart for sixty-eight GeneXpert MTB/RIF Ultra-positive, mycobacterial culture-negative human sputum samples processing and PCR testing for mycobacterial identification. MTBC: *Mycobacterium tuberculosis* complex.

Upon sputum collection in the Vukuzazi study, all sputum specimens (at least 2 ml, not >5 ml) were chemically decontaminated using *N*-acetyl-l-cysteine–sodium hydroxide (NALC-NaOH) for 15 min, concentrated by centrifugation at 3000*g* for 15 min, and the NALC-NaOH supernatants discarded, as previously described [30]. Subsequently, all decontaminated sputum samples were cultured on the conventional liquid mycobacterial culture BACTEC MGIT 960 System (Becton Dickinson, Berkshire, UK), tested for MTBC DNA using the GeneXpert MTB/RIF Ultra qPCR assay (Cepheid, Sunnyvale, California, USA) and parallel 1 ml aliquots were heat-inactivated (45 min at 98 °C) and stored at −80 °C, as previously described [31]. Sixty-eight Ultra-positive, mycobacterial culture-negative (at 42 days incubation), frozen sputum aliquots (1 ml) were made available for this pilot study (Fig. 1 and Supplementary Table 1) [27].

Total DNA was extracted from each 1 ml heat-inactivated sputum aliquot using a modified version of the DNeasy Blood and Tissue kit (Qiagen, Venlo, Limburg, The Netherlands), as detailed in prior literature [32]. Total DNA concentrations were determined with the Qubit 1× dsDNA High Sensitivity Assay kit (Thermo Fisher Scientific), according to the manufacturer's instructions. Samples were tested using a universal bacterial *16S* PCR assay to check that DNA could be amplified, as previously described [33]. Controls included: 1) DNA extraction controls, and 2) PCR amplification controls. All controls were included during each PCR and subsequent sequencing events.

### 2.3 Mycobacteria spp. housekeeping genes PCR amplification and sanger sequencing

To identify samples containing adequate amount of MTBC DNA for subsequent PCR genotyping analyses, we conducted separate amplifications and Sanger sequencing of two specific housekeeping genes belonging to the *Mycobacterium* genus, namely *hsp65* (439 bp) and *rpoB* (764 bp), referred to as the Genus PCR (Table 1) [18]. This process was carried out for each DNA extract, following the protocols described above and previously outlined [18]. Amplicons were submitted to Stellenbosch University's Central Analytical Facility (CAF) for Sanger sequencing. Pairwise sequence alignments were conducted using A plasmid Editor (ApE; Version 3.1.3), and consensus sequences were subsequently analysed with the National Centre for Biotechnology Information (NCBI) nucleotide Basic Local Alignment Search Tool (BLASTn) to identify matching sequences within the NCBI database. *Mycobacteria* spp. that met the criterion of sharing >98% identity with MTBC reference sequences in consensus target sequences, were chosen for subsequent investigations. Sputum samples that yielded positive results on both the Ultra assay and the Genus PCR targeting *hsp65* and *rpoB* were designated as the “MTBC-positive subgroup”. This subgroup was the exclusive focus of subsequent speciation analyses throughout the remainder of the study.

**Table 1:** Amplification product sizes indicating the presence or absence of genomic regions of difference (RD) in three different *Mycobacterium tuberculosis* complex (MTC) members.

	MTBC members.		
RD	*M. tuberculosis*	*M. bovis*	*M. bovis* BCG^a^
1	Present (146 bp)	Present (146 bp)	Absent (196 bp)
4	Present (172 bp)	Absent (268 bp)	Absent (268 bp)
9	Present (235 bp)	Absent (108 bp)	Absent (108 bp)
12	Present (369 bp)	Absent (306 bp)	Absent (306 bp)

a BCG: Bacilli Calmette-Guérin.

### 2.4 MTBC-positive subgroup species identification by region of difference (RD) PCR and spoligotyping

In brief, the Region of Difference (RD) PCR (RD-PCR) was performed on all samples targeting specific regions, including RD1, RD4, RD9, and RD12 following established protocols [19]. Based on the amplification of these 4 regions, samples were classified as either containing *M. bovis* DNA, *M. tuberculosis* DNA or having RD-PCR indeterminant results (Table 1). For samples that indicated the presence of RD1 region (146 bp) alongside several other faint bands specifically for RD4, RD9, and RD12, were defined as the RD-PCR indeterminant subgroup. Furthermore, spoligotyping of all samples from the MTBC-positive subgroup was conducted following the established protocol described by Kamerbeek in 1997 [34].

### 2.5 Single nucleotide polymorphism (SNP) level confirmation of *M. bovis* by targeting the *gyrB* gene

To confirm the presence of *M. bovis* DNA at SNP level, two *gyrB* PCRs (*gyrB1*–144 bp and *gyrB2*–107 bp) were performed on all samples previously identified as *M. bovis*-positive by RD-PCR or spoligotyping. Furthermore, the RD-PCR indeterminant subgroup were also investigated. To differentiate *M. bovis* and *M. bovis* BCG, the presence of the virulent RD1 region was used.

The *gyrB* PCRs were designed to target specific positions within the *gyrB* gene (base pairs 675, 756, 1410, 1437, 1440, and 1450), as previously outlined [21]. This was followed by PCR amplicon sequencing utilizing the MinION mk1C device (Oxford Nanopore Technologies plc, Oxford, UK) and bioinformatically analysed as previously described [35,36]. Each pooled amplicon sample was individually barcoded: Barcode numbers 1 to 10 assigned to samples and barcode 13 onward allocated to controls.

## 3 Results

### 3.1 The vukuzazi TB survey ultra and mycobacterial culture results

Of the 68 GeneXpert Ultra-positive, MGIT-negative sputum samples, GeneXpert semi-quantitative values on the screening specimen were “TRACE DETECTED” in 41 (60.3%), “MTB DETECTED VERY LOW” in 19 (27.9%) and “MTB DETECTED LOW” in 8 (11.8%) (Supplementary Table 1) [27].

### 3.2 MTBC confirmation by *Mycobacteria* spp. housekeeping genes PCR amplification, sanger sequencing

Presence of MTBC DNA was confirmed in 20/68 (29.4%) GeneXpert Ultra-positive sputum samples, based on amplicon Sanger sequencing of the *hsp65* and *rpoB* genes (Genus PCR), respectively (Supplementary Table 1). These samples were defined as the MTBC-positive subgroup (*n* = 20) for further analyses (Table 2 and Supplementary Table 1).

**Table 2:** Results for Region of Difference PCRs (RD-PCR), spoligotypes and *gyrB* amplicon sequencing of DNA extracted from human sputum samples (*n* = 20) that were GeneXpert MTB/RIF Ultra positive and Genus PCR (*hsp65* and *rpoB*) positive for MTBC DNA, and mycobacterial culture negative.

No.^a^	GeneXpert Ultra^b^	Genus PCR^c^	RD-PCR^d^	Spoligotypes^e^	*gyrB* confirmation^f^	Study outcome^g^
13	TRACE	MTBC	Indeterminant^h^	*SB0130*	*M. bovis* (#1)	*M. bovis*
17	LOW	MTBC	Indeterminant^h^	Negative	*M. bovis* (#2)	*M. bovis*
20	VERY LOW	MTBC	*M. bovis*	*SB1474*	*M. bovis* (#3)	*M. bovis*
26	TRACE	MTBC	Indeterminant^h^	Negative	*M. bovis* (#4)	*M. bovis*
31	VERY LOW	MTBC	Indeterminant^h^	*SB0130*	*M. bovis* (#5)	*M. bovis*
36	TRACE	MTBC	Indeterminant^h^	Negative	*M. bovis* (#6)	*M. bovis*
39	VERY LOW	MTBC	*M. bovis*	Negative	*M. bovis* (#7)	*M. bovis*
45	VERY LOW	MTBC	*M. bovis*	Negative	*M. bovis* (#8)	*M. bovis*
48	VERY LOW	MTBC	Indeterminant^h^	*SB1474*	*M. bovis* (#9)	*M. bovis*
55	TRACE	MTBC	Indeterminant^h^	Negative	*M. bovis* (#10)	*M. bovis*
2	VERY LOW	MTBC	*M. tuberculosis*	Negative	n.d.	*M. tuberculosis*
6	VERY LOW	MTBC	*M. tuberculosis*	Negative	n.d.	*M. tuberculosis*
14	TRACE	MTBC	*M. tuberculosis*	Negative	n.d.	*M. tuberculosis*
41	VERY LOW	MTBC	*M. tuberculosis*	Negative	n.d.	*M. tuberculosis*
58	TRACE	MTBC	*M. tuberculosis*	Negative	n.d.	*M. tuberculosis*
60	LOW	MTBC	*M. tuberculosis*	Negative	n.d.	*M. tuberculosis*
66	TRACE	MTBC	*M. tuberculosis*	Negative	n.d.	*M. tuberculosis*
10	TRACE	MTBC	Negative	Negative	n.d.	Negative
40	VERY LOW	MTBC	Negative	Negative	n.d.	Negative
51	TRACE	MTBC	Negative	Negative	n.d.	Negative

a De-identified patient study identity numbers.

b GeneXpert MTB/RIF Ultra assay initial field results for MTBC DNA from the Vukuzazi study.

c Genus PCR (*hsp65* and *rpoB*) assay results for MTBC DNA.

d Region of Difference PCR for MTBC speciation.

e Spoligotyping or spacer oligonucleotide typing.

f SNP-level verification of the presence of *M. bovis*/*M. bovis* BCG and or other MTBC through Oxford Nanopore Technology amplicon sequencing with sample barcode numbers indicated in parenthesis.

g Final study outcome after considering each sample's RD-PCR, spoligotyping, and *gyrB* results.

h RD1 region present (146 bp band) indicative of MTBC members, with multiple additional faint RD4, RD9 and RD12 bands at various sizes.

### 3.3 MTBC speciation by RD-PCR and spoligotyping

From the MTBC-positive subgroup (n = 20), the RD-PCR identified; a) 7/20 (35%) as *M. tuberculosis*, b) 3/20 (15%) as *M. bovis*, c) 7/20 (35%) as RD-PCR indeterminant and 3/20 (15%) as RD-PCR negative (Table 2). Moreover, 4 samples were successfully spoligotyped; 2 were identified as *SB0130* (2 RD-PCR indeterminants) and 2 identified as *SB1474* (1 *M. bovis* and 1 RD-PCR indeterminant) (Table 2, Table 3). From the spoligotyping results, 3 RD-PCR indeterminant samples were shown to also contain *M. bovis* in addition to the 3 *M. bovis* positive samples identified by RD-PCR, with 4 samples remaining as RD-PCR indeterminant (Table 2). Of the 20 MTBC-positive subgroup, 3/20 (13.6%) samples were negative based on RD-PCR and spoligotypes results (Table 2).

**Table 3:**
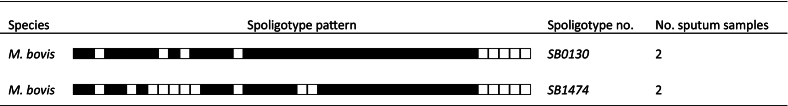
*Mycobacterium bovis* spoligotype patterns identified in 4 of 10 *M. bovis* DNA-positive human sputum samples.

### 3.4 Analysis of *gyrB1* and *gyrB2* gene sequences for *M. bovis* confirmation and RD-PCR indeterminant clarification

Both the *gyrB1* and *gyrB2* gene regions were successfully amplified for all 6 *M. bovis* confirmed samples and the 4 remaining RD-PCR indeterminant samples (Table 2). After filtering reads based on size (50–300 bp) and minimum quality score of 12, consensus sequences were generated for each gene target and sample, based on sequence similarity and length. Mean read quality was 13.5 (SD = 0.09) and mean read length 246 (SD = 3.89). Consensus sequences for *gyrB1* were obtained after combining >23,000 reads per sample (M = 35,993 reads, SD = 11,272) and > 9000 (M = 26,892 reads, SD = 9234) for *gyrB2* (Supplementary QC material). Finally, all consensus sequences were queried against our curated database (consisting of *gyrB* sequences for all MTBC members). The output was evaluated for sequence coverage and percentage identities (Supplementary QC material). All consensus sequences (10 for *gyrB1,* 10 for *gyrB2*) perfectly matched to *M. bovis*/*M. bovis BCG* for the respective genes with coverage and identity of 100% (Supplementary QC material). Additionally, *gyrB1* and *gyrB2* consensus sequences for each sample were aligned against reference *gyrB* sequences of *M. tuberculosis H37Rv* and *M. bovis* AF2122/97, respectively (Supplementary Figure). Notably, the 10 samples (6 *M. bovis* confirmed and 4 RD-PCR indeterminant) appeared to contain *M. bovis* DNA, based on the presence of RD1 region (Table 2 and Supplementary Figure).

### 3.5 Demographics, health status, TB history, symptoms, and radiological findings of people with *M. bovis* DNA in the sputum

The demographic and clinical details of the 10 patients with *M. bovis* DNA identified in their sputum samples are shown in Table 4. The median age was 34.5 years (range 15–68), 3/10 (30%) were male and 7/10 (70%) were female, with a median body mass index (BMI) of 23.41 (range 18.7–34.48)). Seventy percent (7/10) rated themselves in excellent subjective health on the validated quality of life scale (EQ-5D-3L). Fifty percent (5/10) were HIV-positive and on antiretroviral therapy, 2/10 (20%) were active tobacco smokers and 1/10 (10%) endorsed active alcohol drinking. Thirty percent (3/10) endorsed at least one of the WHO TB screening symptoms (cough of any duration, fever, night sweats or unintentional weight loss), 1/10 (10%) were on TB treatment for a concurrent diagnosis of TB at the time of the survey and 5/10 (50%) had a history of previous TB. Fifty percent (5/10) had normal chest radiographs and 5/10 (50%) had abnormal chest radiographs with 2 of these considered by an experienced radiologist to be consistent with active TB and 3 of them abnormal but inconsistent with active TB (Table 4 and Supplementary Table 2).

**Table 4:** Demographics, health status, TB history, symptoms, and radiological findings for patients with sputum identified as positive for *Mycobacterium bovis* DNA (*M. bovis, n* = 10).

No.	This study's outcome^a^	Age	Sex	Weight (kg)	BMI^b^	Health^c^	Current TB^d^	Previous TB^e^	TB household^f^	TB symptoms^g^	X-ray findings^h^	Smoker	Alcohol consumption	HIV
13	*M. bovis*	24	Female	58. 8	20.35	100	No	No	No	No	Normal	No	No	Negative
17	*M. bovis*	68	Female	56	25.92	65	Yes	Yes	No	No	ABN - other	No	No	Negative
20	*M. bovis*	65	Male	95	34.48	100	No	Yes	No	Yes	ABN - other	Yes	No	Positive
26	*M. bovis*	19	Female	78	27.64	100	No	No	No	No	Normal	No	No	Negative
31	*M. bovis*	18	Female	67.3	24.72	100	No	No	NA	No	Normal	No	No	Negative
36	*M. bovis*	15	Female	43.8	22.03	100	No	Yes	Yes	Yes	Normal	No	Yes	Positive
39	*M. bovis*	31	Male	56	18.71	100	No	No	No	No	ABN - TB	Yes	No	Positive
45	*M. bovis*	38	Female	63	21.3	100	No	No	No	No	Normal	No	No	Positive
48	*M. bovis*	60	Male	70	22.09	80	No	Yes	No	Yes	ABN - TB	No	No	Negative
55	*M. bovis*	49	Female	73	32.02	99	No	Yes	No	No	ABN - other	No	No	Positive

a *M. bovis DNA* outcome from this study directly from DNA extracted from decontaminated raw sputum pellets.

b Body mass index: < 18.5 = underweight range; between 18.5 and 24.9 = healthy weight range; 25.0 and 29.9 = overweight; 30.0 or higher = obese.

c Self-reported health state (EQ-5D-3L): Best health is marked 100 (one hundred) top of the scale, and the worst state is marked 0 (zero) at the bottom (EQ-5D-3L: https://euroqol.org/eq-5d-instruments/eq-5d-3l-about/, Version11JAN2022).

d Diagnosed clinically at the time of collection and on TB treatment.

e Previously diagnosed with tuberculosis*.*

f Had at any time lived with someone that had tuberculosis.

g Self-reported TB symptoms: cough, fever, night sweats of any duration or unintended weight loss.

h Radiological evidence of lung abnormalities indicative of TB or other disease.

## 4 Discussion

While sputum samples, traditionally employed for pulmonary TB diagnosis, are not ideal for *M. bovis* detection due to its predilection for extrapulmonary disease, instances of its presence in sputum and lymph nodes are not uncommon [4,13,15,37,38], especially in high-risk areas with HIV and TB co-prevalence and endemic *M. bovis* in wildlife and livestock, like many low and middle-income African countries [14,15,[39], [40], [41]]. These studies hypothesize that viable *M. bovis* in human respiratory samples may result from inhalation of bacilli-laden aerosols during direct contact with infected animals (including carcasses), exposure to contaminated environments, or consumption of contaminated food like unpasteurized milk or cheese [6,8,[42], [43], [44]]. Nevertheless, these studies emphasize the requirement for multi-centre, health facility-based cross-sectional approaches for *M. bovis* detection. They further highlight the importance of employing diverse molecular detection methods, including lymph node samples alongside sputum, and the benefits of enhancing conventional culture methods with pyruvate addition and extended culture incubation periods exceeding 42 days [15,39].

Our study utilized sputum samples and conventional mycobacterial culture (without added pyruvate and an incubation period of 42 days) results. Despite the failure of culture in the parent study, we successfully detected *M. bovis* and *M. tuberculosis* DNA from 10/20 and 7/20 selected sputum samples, respectively, within the MTBC positive subgroup (Table 2). Through spoligotyping, we further identified two *M. bovis* strains (SB*0130* and SB*1474*) in 4/10 *M. bovis* DNA positive samples (Table 2, Table 3) [[45], [46], [47]]. These results further emphasized the constraints of relying solely on traditional mycobacterial culture and stress the significance of integrating multiple diverse molecular techniques with different sensitivities and suitable clinical specimens for a thorough comprehension of zoonotic TB transmission [13,18,20,21,48].

Culture-negative sputum with DNA evidence of *M. bovis* or *M. tuberculosis* may result from: a) residual DNA from cleared infections, indicated by distinct radiographic abnormalities; b) oral contamination from external sources like tainted food or water; c) non-culturable bacteria in incipient disease, as seen in MTBC DNA-positive patients reporting good health, lacking TB history, and normal radiographs [49]; d) generally paucibacillary sputum samples, as suggested by low Ultra results and radiographic TB abnormalities, influenced by excessive pre-culture decontamination; e) the 42-day culture incubation period limitation [46]; or f) culture medium suitability for *M. bovis* (pH 5–6.5, no added casein, pyruvate, or pyrazinamide) [46]. Ultra-positive samples with undetectable MTBC DNA in other PCRs indicate either false-positive Ultra results or values below the lower detection limit in these downstream PCRs (Supplementary Table 1).

**Fig. 2:**
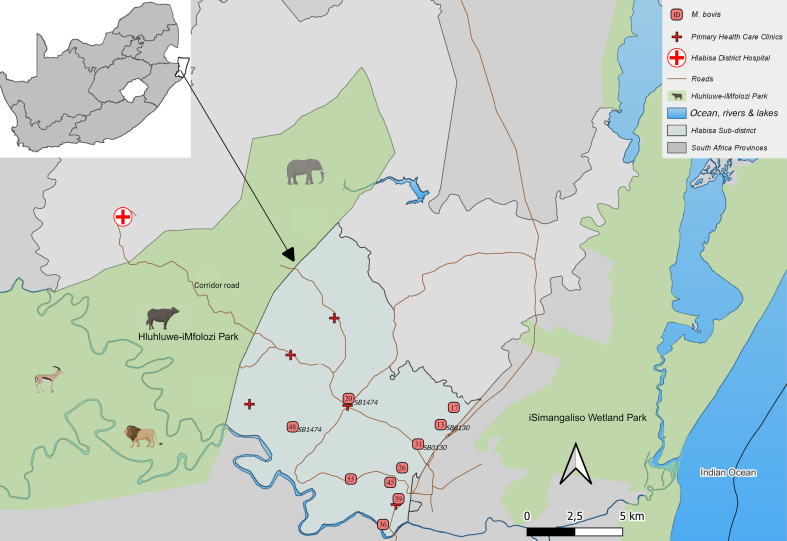
Approximate home locations of 10 patients identified to contain *Mycobacterium bovis* DNA (n = 10, pink dots with patient study identifiers) in their sputum samples. Locations are shown relative to Hluhluwe-iMfolozi Park, iSimangaliso Wetland Park, Primary Health Care Clinics, and Hlabisa District Hospital in Hlabisa Sub-district, KwaZulu-Natal Province, South Africa. (For interpretation of the references to colour in this figure legend, the reader is referred to the web version of this article.)

Previous studies of *M. bovis* in animals from the Hlabisa sub-district provide valuable insights into the genetic diversity of *M. bovis* strains circulating [6,8,9]. These studies revealed a shared spoligotype pattern (SB*0130*) between *M. bovis* isolates from wildlife and communal cattle and a African buffalo-specific spoligotype (SB*1474*) [9]. In our study, the detection of both these spoligotypes in human sputum specimens suggests either recent or historic potential cross-species transmission at the wildlife-livestock-human interface surrounding HiP (Table 3). Moreover, the identification of a *M. bovis* spoligotype only associated with HiP buffalo populations (SB*1474*) [9] in human samples has significant implications for zoonotic transmission. The proximity of communities and their livestock to *M. bovis* endemic HiP (Fig. 2) and the frequent consumption of “bushmeat”, including buffalo meat, from HiP, raises concerns about the potential for spillover of the pathogen directly or indirectly from wildlife to humans [33].

Identification of *M. bovis* strains (*SB0130* and *SB1474*) in human samples highlights the necessity for enhanced surveillance and control in communities with high HIV/AIDS prevalence and significant wildlife-livestock-human interfaces, at risk of zoonotic TB [6,16]. The coexistence of diverse susceptible hosts in shared ecosystems poses control challenges, necessitating a One Health approach. Understanding factors driving cross-species transmission and the genetic diversity of *M. bovis* strains is vital for targeted interventions.

Acknowledging study limitations is crucial. Despite available health data, the absence of host cell-mediated immunological responses, such as those assessed using the QuantiFERON TB Gold Plus test, complicates the assessment of true infection status [4,49]. Heat-inactivation limits all culture methods [48]. The limited sample size prohibits a comprehensive investigation into *M. bovis* strain diversity. Expanding samples to include various extrapulmonary samples and broader geographic areas would enhance epidemiological insights. Speciation PCRs' variation in limit of detection, compared to the Ultra, may underreport the presence of *M. tuberculosis* and *M. bovis* DNA [22]. Future research should explore improved *M. bovis*-specific culture methods, advanced PCR techniques, and whole-genome sequencing of cultured isolates for comprehensive understanding [17,45,46].

Implications extend to tuberculosis control in South Africa, emphasizing the importance of awareness regarding MTBC DNA-positive, Ultra-culture-negative cases near *M. bovis*-endemic regions. Thorough investigations into *M. bovis* presence in respiratory samples are crucial for determining pathogenicity. Insights from wildlife and livestock TB inform interventions for mitigating zoonotic TB. Integrating human and animal health systems is vital, particularly in wildlife-livestock-human interface regions. Control measures should include strategies to reduce raw animal product consumption. [5].

## 5 Conclusions

In conclusion, our pilot study highlights the substantial prevalence of detected *M. bovis* among people residing near HiP in SA, shedding light on the intricate interplay between human, animal, and environmental health. The identification of shared *M. bovis* strains among individuals, cattle, and African buffaloes underscores the potential for cross-species transmission, emphasizing the interconnected nature of health risks in these regions. Moving forward, it is crucial to prioritize heightened surveillance and implement comprehensive, collaborative, and interdisciplinary One Health approaches that integrate veterinary, wildlife, and public health efforts. South African agencies such as the Department of Health, the Department of Agriculture, Land Reform and Rural development, the National Institute for Communicable Diseases, the South African Veterinary Association, and relevant academic institutions must be involved in this effort. This collaborative approach is essential for effectively addressing the complex challenges associated with zTB transmission and promoting the health and well-being of both human, animal and environmental health in these areas.

The following are the supplementary data related to this article.

**Supplementary Fig. 1:**
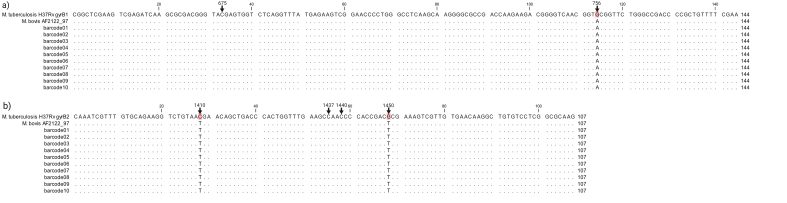
Differentiation of *Mycobacterium tuberculosis* complex based on six different single nucleotide polymorphisms (SNPs). Numbers represent the position of the SNP in relation to the start codon of *gyrB*. **(a)** Two SNPs in *gyrB1* at base pair positions 675, 756 and **(b)** four SNPs in *gyrB2* at base pair positions 1410, 1437, 1440 and 1450 are represented [21]. *Mycobacterium bovis* DNA was confirmed at SNP-level for all 10 sputum samples (6 *M. bovis* confirmed by RD-PCR or Spoligotyping and 4 RD-PCR indeterminant) with no indication of any other underlying MTBC population.

Supplementary material 1 Demographics, Health Status, TB History, Symptoms, and Radiological Findings of people with M. bovis DNA in the sputum. Supplementary material 1

Supplementary material 2 Total amount of sequencing reads for gyrB1 and gyrB2 M. bovis confirmation. Supplementary material 2

Supplementary material 3 Supplementary Figure 1 description. Supplementary material 3

Supplementary material 4 Vukuzazi Team: Staff who significantly contributed to the implementation and conduct of Vukuzazi. Supplementary material 4

Supplementary material 5 PDF of the MinKNOW Run Report using Oxford Nanopore Technologies mk1c device and R10.4.1 flow cell for M. bovis confirmation. Supplementary material 5

Supplementary material 6 Confirmation of Publication and Licensing Rights for Figure 2. Supplementary material 6

Supplementary material 7 European Nucleotide Archive specific project code and accession numbers for all sequences generated. Supplementary material 7

Supplementary material 8 MinKNOW Run Report using Oxford Nanopore Technologies mk1c device and R10.4.1 flow cell for M. bovis confirmation. Supplementary material 8

Supplementary material 9 Vukuzazi Team: Staff who significantly contributed to the implementation and conduct of Vukuzazi. Supplementary material 9

## Funding

The project is funded by a) Wellcome Foundation [grant #222941/Z/21/Z], b) the 10.13039/501100000780European Union supported by the Global Health EDCTP3 Joint Undertaking and its members [Project 101103171], c) 10.13039/501100001322South African Medical Research Council Centre for TB Research, d) National Research Foundation South African Research Chair Initiative [grant #86949], e) Wellcome Strategic Core award [201433/Z/16/A] and f) Burroughs Wellcome Pathogenesis of Infectious Disease Fellowship [1022002, EBW]. Views and opinions expressed are however those of the author(s) only and do not necessarily reflect those of the European Union, Global Health EDCTP3 Joint Undertaking, Wellcome, SAMRC CTR or NRF. None can be held responsible for them.

## Ethics statement

Ethical approval for the study was obtained from the Ethics Committees of the University of KwaZulu-Natal (BE560/17), London School of Hygiene & Tropical Medicine (#14722), the Partners Institutional Review Board (2018P001802), University of Alabama at Birmingham (#300007237) and Stellenbosch University (BES-2023-22,561; MTA- S008884).

## CRediT authorship contribution statement

**Wynand J. Goosen:** Conceptualization, Data curation, Formal analysis, Funding acquisition, Investigation, Methodology, Resources, Visualization, Writing – original draft, Writing – review & editing. **Sashen Moodley:** Project administration, Writing – review & editing. **Giovanni Ghielmetti:** Data curation, Formal analysis, Visualization, Writing – review & editing. **Yumna Moosa:** Writing – review & editing. **Thando Zulu:** Project administration, Writing – review & editing. **Theresa Smit:** Project administration, Writing – review & editing. **Leanie Kleynhans:** Conceptualization, Methodology, Writing – review & editing. **Tanya J. Kerr:** Methodology, Project administration, Writing – review & editing. **Elizabeth M. Streicher:** Methodology, Resources, Writing – review & editing. **Willem A. Hanekom:** Resources, Supervision, Writing – review & editing. **Robin M. Warren:** Conceptualization, Funding acquisition, Methodology, Resources, Supervision, Validation, Writing – review & editing. **Emily B. Wong:** Conceptualization, Supervision, Writing – review & editing. **Michele A. Miller:** Conceptualization, Funding acquisition, Resources, Supervision, Writing – review & editing.

## Declaration of competing interest

All authors declare no competing financial and/or non-financial interests in relation to the work described.

## Data Availability

All sequencing data (including DNA extraction, PCR, and sequencing controls) were uploaded to the European Nucleotide Archive (ENA) and the specific project code and accession numbers are available as supplementary material.

## References

[1] Sichewo P.R., Etter E.M.C., Michel A.L. (Jan. 2020). Wildlife-cattle interactions emerge as drivers of bovine tuberculosis in traditionally farmed cattle. Prev. Vet. Med..

[2] Bernitz N. (2021). Review of diagnostic tests for detection of *Mycobacterium bovis* infection in south African wildlife. Front. Vet. Sci..

[3] Michel A.L., Müller B., van Helden P.D. (Jan. 2010). *Mycobacterium bovis* at the animal–human interface: a problem, or not?. Vet. Microbiol..

[4] Müller B. (Jun. 2013). Zoonotic *Mycobacterium bovis*-induced tuberculosis in humans. Emerg. Infect. Dis..

[5] Gortázar C., de la Fuente J., Perelló A., Domínguez L. (Sep. 2023). Will we ever eradicate animal tuberculosis?. Ir. Vet. J..

[6] Sichewo P.R., Hlokwe T.M., Etter E.M.C., Michel A.L. (Mar. 2020). Tracing cross species transmission of *Mycobacterium bovis* at the wildlife/livestock interface in South Africa. BMC Microbiol..

[7] Palmer M.V. (Nov. 2013). *Mycobacterium bovis*: characteristics of wildlife reservoir hosts. Transbound. Emerg. Dis..

[8] Sichewo P.R., Michel A.L., Musoke J., Etter E.M.C. (Jul. 2019). Risk factors for zoonotic tuberculosis at the wildlife-livestock-human interface in South Africa. Pathogens.

[9] Hlokwe T.M. (Jun. 2011). Molecular characterisation of *Mycobacterium bovis* isolated from African buffaloes (*Syncerus caffer*) in Hluhluwe-iMfolozi park in KwaZulu-Natal, South Africa. Onderstepoort J. Vet. Res..

[10] Leung Soo C., Pant Pai N., Bartlett S.J., Esmail A., Dheda K., Bhatnagar S. (Jan. 2023). Socioeconomic factors impact the risk of HIV acquisition in the township population of South Africa: A Bayesian analysis. PLOS Glob Public Health.

[11] Claro-Almea F.E., Delgado-Noguera L.A., Motaban A., España M., de Waard J.H. (Oct. 2020). A rare case of spinal tuberculosis due to *Mycobacterium bovis*. Is zoonotic tuberculosis underdiagnosed?. IDCases.

[12] Ajudua F.I., Mash R.J. (Oct. 2020). Implementing active surveillance for TB—the views of managers in a resource limited setting, South Africa. PLoS One.

[13] van der Westhuizen C.G., Burt F.J., van Heerden N., van Zyl W., Anthonissen T., Musoke J. (2023). Prevalence and occupational exposure to zoonotic diseases in high-risk populations in the Free State Province, South Africa. Front. Microbiol..

[14] Sanou A. (Oct. 2014). *Mycobacterium bovis* in Burkina Faso: epidemiologic and genetic links between human and cattle isolates. PLoS Negl. Trop. Dis..

[15] Ayalew S. (2023). Zoonotic tuberculosis in a high bovine tuberculosis burden area of Ethiopia. Front. Public Health.

[16] Mtetwa H.N., Amoah I.D., Kumari S., Bux F., Reddy P. (Aug. 2023). Exploring the role of wastewater-based epidemiology in understanding tuberculosis burdens in Africa. Environ. Res..

[17] Rossi G. (2023). Unraveling the epidemiology of *Mycobacterium bovis* using whole-genome sequencing combined with environmental and demographic data. Front. Vet. Sci..

[18] Goosen W.J., Clarke C., Kleynhans L., Kerr T.J., Buss P., Miller M.A. (Jun. 2022). Culture-independent PCR detection and differentiation of *mycobacteria spp*. in antemortem respiratory samples from African elephants (*Loxodonta Africana*) and rhinoceros (*Ceratotherium Simum*, *Diceros Bicornis*) in South Africa. Pathogens.

[19] Warren R.M. (Jul. 2006). Differentiation of *Mycobacterium tuberculosis* complex by PCR amplification of genomic regions of difference. Int. J. Tuberc. Lung Dis..

[20] Goosen W.J. (Sep. 2020). The Xpert MTB/RIF ultra assay detects *Mycobacterium tuberculosis* complex DNA in white rhinoceros (*Ceratotherium simum*) and African elephants (*Loxodonta africana*). Sci. Rep..

[21] Landolt P., Stephan R., Stevens M.J.A., Scherrer S. (2019). Three-reaction high-resolution melting assay for rapid differentiation of *Mycobacterium tuberculosis* complex members. MicrobiologyOpen.

[22] Chakravorty S. (Sep. 2017). The new Xpert MTB/RIF ultra: improving detection of *Mycobacterium tuberculosis* and resistance to rifampin in an assay suitable for point-of-care testing. mBio.

[23] Hlokwe T.M., Mogano R.M. (Apr. 2020). Utility of xpert® MTB/RIF ultra-assay in the rapid diagnosis of bovine tuberculosis in wildlife and livestock animals from South Africa. Prev. Vet. Med..

[24] Zhou L., Zou X., Hu Q., Hua H., Qi Q. (Sep. 2023). Determination of the diagnostic accuracy of nanopore sequencing using bronchoalveolar lavage fluid samples from patients with sputum-scarce pulmonary tuberculosis. J. Infect. Chemother..

[25] Yang J. (Sep. 2023). Accuracy of Nanopore sequencing as a diagnostic assay for pulmonary tuberculosis versus smear, culture and Xpert MTB/RIF: a head-to-head comparison. Trop. Med. Infect. Dis..

[26] Lessells R.J., Mutevedzi P.C., Cooke G.S., Newell M.L. (Mar. 2011). Retention in HIV care for individuals not yet eligible for antiretroviral therapy: rural KwaZulu-Natal, South Africa. J. Acquir. Immune Defic. Syndr..

[27] Gunda R. (Nov. 2021). Cohort profile: the Vukuzazi (‘wake up and know yourself’ in isiZulu) population science programme. Int. J. Epidemiol..

[28] Davey S. (Jul. 2023). Challenges to the control of *Mycobacterium bovis* in livestock and wildlife populations in the south African context. Ir. Vet. J..

[29] Otchere I.D. (Mar. 2019). Molecular epidemiology and whole genome sequencing analysis of clinical *Mycobacterium bovis* from Ghana. PLoS One.

[30] Dippenaar A. (Jan. 2022). Optimizing liquefaction and decontamination of sputum for DNA extraction from *Mycobacterium tuberculosis*. Tuberculosis (Edinb.).

[31] Wong E.B. (Jun. 2021). Convergence of infectious and non-communicable disease epidemics in rural South Africa: a cross-sectional, population-based multimorbidity study. Lancet Glob. Health.

[32] Kerr T.J. (Jan. 2023). Detection of elephant Endotheliotropic herpesvirus (EEHV) in free-ranging African elephants (*Loxodonta Africana*) in the Kruger National Park, South Africa. J. Wildl. Dis..

[33] Van der Merwe M., Michel A.L. (Sep. 2010). An investigation of the effects of secondary processing on *Mycobacterium spp*. in naturally infected game meat and organs. J. S. Afr. Vet. Assoc..

[34] Kamerbeek J. (Apr. 1997). Simultaneous detection and strain differentiation of *Mycobacterium tuberculosis* for diagnosis and epidemiology. J. Clin. Microbiol..

[35] Ghielmetti G. (2023). Advancing animal tuberculosis surveillance using culture-independent long-read whole-genome sequencing. Front. Microbiol..

[36] Vierstraete A.R., Braeckman B.P. (Feb. 2022). Amplicon_sorter: a tool for reference-free amplicon sorting based on sequence similarity and for building consensus sequences. Ecol. Evol..

[37] (2024). Fact Sheets | General | *Mycobacterium bovis* (Bovine Tuberculosis) in Humans | TB | CDC. https://www.cdc.gov/tb/publications/factsheets/general/mbovis.htm.

[38] Malama S., Munyeme M., Mwanza S., Muma J.B. (Sep. 2014). Isolation and characterization of non-tuberculous mycobacteria from humans and animals in Namwala District of Zambia. BMC. Res. Notes.

[39] Katale B.Z. (Jun. 2017). Isolation and potential for transmission of *Mycobacterium bovis* at human-livestock-wildlife interface of the Serengeti ecosystem, northern Tanzania. Transbound. Emerg. Dis..

[40] Ghariani A. (Dec. 2015). Diagnosis of lymph node tuberculosis using the GeneXpert MTB/RIF in Tunisia. Int. J. Mycobacteriol..

[41] Abakar M.F. (Feb. 2017). Transmission dynamics and elimination potential of zoonotic tuberculosis in Morocco. PLoS Negl. Trop. Dis..

[42] Thoen C.O., Lobue P.A., Enarson D.A., Kaneene J.B., de Kantor I.N. (2009). Tuberculosis: a re-emerging disease in animals and humans. Vet. Ital..

[43] Ayele W.Y., Neill S.D., Zinsstag J., Weiss M.G., Pavlik I. (Aug. 2004). Bovine tuberculosis: an old disease but a new threat to Africa. Int. J. Tuberc. Lung Dis..

[44] Allen A.R., Ford T., Skuce R.A. (2021). Does *Mycobacterium tuberculosis var. bovis* survival in the environment confound bovine tuberculosis control and eradication? A literature review. Vet. Med. Int..

[45] Gallagher J., Horwill D.M. (Aug. 1977). A selective oleic acid albumin agar medium for the cultivation of *Mycobacterium bovis*. J. Hyg. (Lond.).

[46] Keating L.A. (Apr. 2005). The pyruvate requirement of some members of the *Mycobacterium tuberculosis* complex is due to an inactive pyruvate kinase: implications for in vivo growth. Mol. Microbiol..

[47] Sung J. (Oct. 2023). Evidence for tuberculosis in individuals with Xpert ultra ‘trace’ sputum during screening of high-burden communities. Clin. Infect. Dis..

[48] Clarke C., Cooper D.V., Miller M.A., Goosen W.J. (Feb. 2022). Detection of *Mycobacterium tuberculosis* complex DNA in oronasal swabs from infected African buffaloes (*Syncerus caffer*). Sci. Rep..

[49] Drain P.K. (Oct. 2018). Incipient and subclinical tuberculosis: a clinical review of early stages and progression of infection. Clin. Microbiol. Rev..

