# Supplementary material for: Identification and molecular characterization of Mycobacterium bovis DNA in GeneXpert® MTB/RIF ultra-positive, culture-negative sputum from a rural community in South Africa
Source: One Health. 2024 Mar 3;18:100702. doi: 10.1016/j.onehlt.2024.100702 (PMC10937233; doi:10.1016/j.onehlt.2024.100702)
Supplement: Supplementary material 4 — Vukuzazi Team: Staff who significantly contributed to the implementation and conduct of Vukuzazi. [file mmc5.docx]

Supplementary Figure Differentiation of Mycobacterium tuberculosis complex based on six different single nucleotide polymorphisms (SNPs). Numbers represent the position of the SNP in relation to the start codon of gyrB. (a) Two SNPs in gyrB1 at base pair positions 675, 756 and (b) four SNPs in gyrB2 at base pair positions 1410, 1437, 1440 and 1450 are represented[21].  Mycobacterium bovis DNA was confirmed at SNP-level for all 10 sputum samples (6 M. bovis confirmed by RD-PCR or Spoligotyping and 4 RD-PCR indeterminant) with no indication of any other underlying MTBC population.
