# Supplementary material for: Identification and molecular characterization of Mycobacterium bovis DNA in GeneXpert® MTB/RIF ultra-positive, culture-negative sputum from a rural community in South Africa
Source: One Health. 2024 Mar 3;18:100702. doi: 10.1016/j.onehlt.2024.100702 (PMC10937233; doi:10.1016/j.onehlt.2024.100702)
Supplement: Supplementary material 5 — PDF of the MinKNOW Run Report using Oxford Nanopore Technologies mk1c device and R10.4.1 flow cell for M. bovis confirmation. [file mmc6.docx]

**Vukuzazi Team: Staff who significantly contributed to the implementation and conduct of Vukuzazi.**

* Denotes team members who were closely involved with the design, implementation and oversight of Vukuzazi.

| **Name** | **Role** |
| --- | --- |
| *Deenan Pillay | Principal Investigator (2017-2019) |
| *Willem Hanekom | Principal Investigator (2019-present) |
| *Emily Wong | Co-Principal Investigator |
| *Mark Siedner | Co-Principal Investigator |
| *Olivier Koole^2,6^ | Co-Principal Investigator (2017-2019) |
| *Thumbi Ndung’u | Co-investigator |
| *Thandeka Khoza | Co-investigator (2019-present) |
| *Kobus Herbst | Co-investigator |
| *Kathy Baisley | Co-investigator |
| *Janet Seeley | Co-investigator |
| *Alison Grant | Co-investigator |
| *Resign Gunda | Programme Manager |
| *Ashmika Surujdeen | Study Coordinator |
| *Theresa Smit | Head: Diagnostic Research |
| *Dickman Gareta | Head: Research Data Management |
| *Day Munatsi | Head: Research Data Systems |
| *Ngcebo Mhlongo | Study Physician |
| *Sanah Bucibo | Lead Nurse |
| *Tshwaraganang Modise | Research Data Manager |
| *Stephen Olivier | Statistician |
| *Gregory Ording-Jespersen | Laboratory Data Supervisor |
| *Innocentia Mpofana | Diagnostic Laboratory Manager |
| *Jaco Dreyer | Senior Research Data Manager |
| *Siyabonga Nxumalo | Research Data Manager |
| *Khadija Khan | Biorepository Manager |
| *Zizile Sikhosana | Somkhele Laboratory Supervisor |
| *Sashen Moodley | Microbiology Laboratory Supervisor |
| *Hollis Shen | Head: Exploratory Research Division |
| Kennedy Nyamande | Pulmonology Consultant |
| Mosa Suleman | Pulmonology Consultant |
| Jaikrishna Kalideen | Radiologist |
| Ramesh Jackpersad | Radiologist |
| Kgaugelo Moropane | Radiographer |
| Boitsholo Mfolo | Radiographer |
| Khabonina Malomane | Radiographer |
| Hlolisile Khumalo | Nursing Manager |
| Nompilo Buthelezi | Training Coordinator |
| Nozipho Mbonambi | Professional Nurse |
| Hloniphile Ngubane | Professional Nurse |
| Thokozani Simelane | Professional Nurse |
| Khanyisani Buthelezi | Professional Nurse |
| Sphiwe Ntuli | Professional Nurse |
| Nombuyiselo Zondi | Professional Nurse |
| Siboniso Nene | Professional Nurse |
| Bongumenzi Ndlovu | Enrolled Nurse |
| Talente Ntimbane | Enrolled Nurse |
| Mbali Mbuyisa | Enrolled Nurse |
| Xolani Mkhize | Enrolled Nurse |
| Melusi Sibiya | Enrolled Nurse |
| Ntombiyenkosi Ntombela | Enrolled Nurse |
| Mandisi Dlamini | Enrolled Nurse |
| Hlobisile Chonco | Enrolled Nurse |
| Hlengiwe Dlamini | Enrolled Nurse |
| Doctar Mlambo | Enrolled Nurse |
| Nonhlanhla Mzimela | Enrolled Nurse |
| Zinhle Buthelezi | Enrolled Nurse |
| Zinhle Mthembu | Enrolled Nurse |
| Thokozani Bhengu | Enrolled Nurse |
| Sandile Mthembu | Enrolled Nurse |
| Phumelele Mthethwa | Enrolled Nurse |
| Zamashandu Mbatha | Enrolled Nurse |
| Welcome Petros Mthembu | Enrolled Nurse |
| Anele Mkhwanazi | Clinical Research Assistant Supervisor |
| Mandlakayise Zikhali | Clinical Research Assistant Supervisor |
| Phakamani Mkhwanazi | Clinical Research Assistant |
| Ntombiyenhlanhla Mkhwanazi | Clinical Research Assistant |
| Rose Myeni | Clinical Research Assistant |
| Fezeka Mfeka | Clinical Research Assistant |
| Hlobisile Gumede | Clinical Research Assistant |
| Nonceba Mfeka | Clinical Research Assistant |
| Ayanda Zungu | Clinical Research Assistant |
| Hlobisile Gumede | Clinical Research Assistant |
| Nonhlanhla Mfekayi | Clinical Research Assistant |
| Smangaliso Zulu | Clinical Research Assistant |
| Mzamo Buthelezi | Clinical Research Assistant |
| Senzeni Mkhwanazi | Clinical Research Assistant |
| Mlungisi Dube | Clinical Research Assistant |
| Philippa Mathews | Clinical Governance |
| Siphephelo Dlamini | AHRI Nursing Manager |
| Hosea Kambonde | IT Systems Developer |
| Lindani Mthembu | Information Technology Assistant |
| Seneme Mchunu | Information Technology Assistant |
| Sibahle Gumbi | Research Admin Assistant |
| Tumi Madolo | Research Data Manager |
| Thengokwakhe Nkosi | Driver |
| Sibusiso Mkhwanazi | Driver |
| Sibusiso Nsibande | Driver |
| Mpumelelo Steto | Driver |
| Sibusiso Mhlongo | Driver |
| Velile Vellem | Driver |
| Pfarelo Tshivase | Driver |
| Jabu Kwinda | Driver |
| Bongani Magwaza | General Worker |
| Siyabonga Nsibande | General Worker |
| Skhumbuzo Mthombeni | General Worker |
| Sphiwe Clement Mthembu | General Worker |
| Antony Rapulana | Laboratory Technologist |
| Jade Cousins | Laboratory Technologist |
| Thabile Zondi | Laboratory Technologist |
| Nagavelli Padayachi | Laboratory Technologist |
| Freddy Mabetlela | Laboratory Technologist |
| Simphiwe Ntshangase | Laboratory Technician/LIMS Administrator |
| Nomfundo Luthuli | Laboratory Technician |
| Sithembile Ngcobo | Laboratory Technologist |
| Kayleen Brien | Laboratory Technologist |
| Sizwe Ndlela | Laboratory Technician |
| Nomfundo Ngema | Laboratory Technician |
| Nokukhanya Ntshakala | Laboratory Technician |
| Anupa Singh | Laboratory Technician |
| Rochelle Singh | Laboratory Technician |
| Logan Pillay | Laboratory Technician |
| Kandaseelan Chetty | Laboratory Technician |
| Ashentha Govender | Laboratory Technician |
| Pamela Ramkalawon | Laboratory Research Technician |
| Nondumiso Mabaso | Laboratory Intern |
| Kimeshree Perumal | Laboratory Intern |
| Senamile Makhari | Biorepository Laboratory Technician |
| Nondumiso Khuluse | Biorepository Laboratory Technician |
| Nondumiso Zitha | Biorepository Research Assistant |
| Hlengiwe Khathi | Biorepository Research Assistant |
| Mbuti Mofokeng | Clinical Specimen Driver/Laboratory Assistant |
| Nomathamsanqa Majozi | Public Engagement |
| Nceba Gqaleni | Public Engagement |
| Hannah Keal | Communications |
| Phumla Ngcobo | Communications |
| Costa Criticos | Operational Oversight |
| Raynold Zondo | Operational Oversight |
| Dilip Kalyan | Operational Oversight |
| Clive Mavimbela | Operational Oversight |
| Anand Ramnanan | Procurement |
| Sashin Harilall | Grants Office |
