# Supplementary material for: Identification and molecular characterization of Mycobacterium bovis DNA in GeneXpert® MTB/RIF ultra-positive, culture-negative sputum from a rural community in South Africa
Source: One Health. 2024 Mar 3;18:100702. doi: 10.1016/j.onehlt.2024.100702 (PMC10937233; doi:10.1016/j.onehlt.2024.100702)
Supplement: Supplementary material 6 — Confirmation of Publication and Licensing Rights for Figure 2. [file mmc7.pdf]

MinION Mk1C (MC-114017) Final report

07 Aug 23, 08:16 — 08 Aug 23, 00:42 · 20230807_AHRI5sigsputumzTB · no_sample · MC-114017
Protocol run ID: f8863b05-eca8-4225-ac76-2fc426a8f9f8

Run summary | Run configuration | Sequence output | Run health | Run log

Run summary

DATA OUTPUT

Estimated bases	Data produced
3.37 Gb	90.43 GB
**Reads generated**	**Estimated N50**
5.6 M	873

RUN DURATION

Elapsed time

80 hours of 80 hours

Run status

Stopped by user

View unit abbreviations used in this report

BASECALLING

Reads called	Bases called (min Q score: 9) Pass	Fail
100%	2.9 Gb	335.32 Mb

Run configuration

RUN SETUP

Flow cell type	FLO-MIN114
Flow cell type alias	FLO-MIN114
Flow cell ID	FAW08908
Kit type	SQK-NBD114-96

RUN SETTINGS

Specified run length	80 hrs
Active channel selection	On
Pore scan freq.	1.5 hrs
Reserved pores	On
Minimum read length	20 bp
Read splitting	On
Basecalling	High-accuracy model, 260 bps
Modified basecalling	Off
Trim barcodes	Off
Mid-read barcode filtering	Off

DATA OUTPUT SETTINGS

FAST5 output	vbz_compress
FAST5 reads per file	4000
FASTQ output	gzip_compress
FASTQ reads per file	4000
BAM output	Off
Bulk file output	Off
Data location	/data/./20230807_AHRI5sigsputumzTB/no_sample/20230807_0816_MC-114017_FAW08908_f8863b05

SOFTWARE VERSIONS

MinKNOW	22.12.5
Bream	7.4.8
Configuration	5.4.7
Guppy	6.4.6
MinKNOW Core	5.4.3

Sequence output

READ LENGTHS · OUTLIERS REMOVED

The read length graph shows the total number of bases vs the read length. The longest 1% of strands are classified as outliers, and excluded to allow focus on the main body of data.

Legend

Basecalled — Estimated

Estimated N50	% Basecalled
873	100%

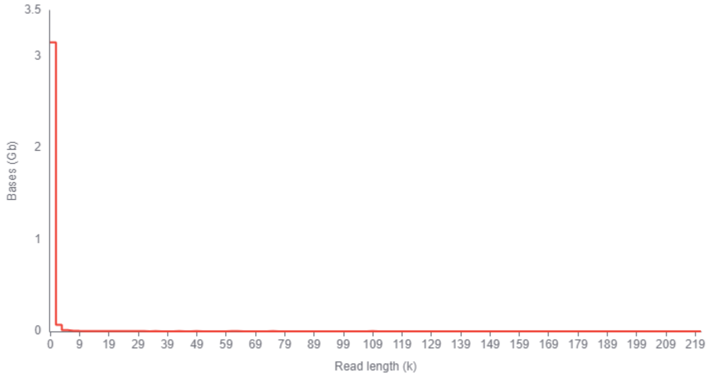

OUTLIERS

The longest 1% of strands are classified as outliers, and aggregated into groups to show their relative amounts.

Read length (kb)	Aggregated reads (Mb)
0 - 128	28.31
128 - 256	0.15
256 - 384	*None*
384 - 492	0.5

BARCODED READS

The total number of bases for each barcode is calculated and displayed below.

Total bases (Gb)	Barcodes
0–1	barcode01 (Reads: 270312) barcode02 (Reads: 279151) barcode03 (Reads: 258272) barcode04 (Reads: 299275)<br>barcode05 (Reads: 327746) barcode06 (Reads: 313714) barcode07 (Reads: 330402) barcode08 (Reads: 320241)<br>barcode09 (Reads: 324697) barcode10 (Reads: 337639) barcode11 (Reads: 638903) barcode12 (Reads: 302724) barcode13 (Reads: 6537)<br>barcode14 (Reads: 1) barcode15 (Reads: 4) barcode16 (Reads: 1) barcode17 (Reads: 2) barcode18 (Reads: 7) barcode19 (Reads: 0)<br>barcode20 (Reads: 1) barcode21 (Reads: 3) barcode22 (Reads: 8) barcode23 (Reads: 0) barcode24 (Reads: 4) barcode25 (Reads: 1)<br>barcode26 (Reads: 2) barcode27 (Reads: 2) barcode28 (Reads: 2) barcode29 (Reads: 3) barcode30 (Reads: 0) barcode31 (Reads: 1)<br>barcode32 (Reads: 6) barcode33 (Reads: 4) barcode34 (Reads: 4) barcode35 (Reads: 1) barcode36 (Reads: 0) barcode37 (Reads: 0)<br>barcode38 (Reads: 0) barcode39 (Reads: 2) barcode40 (Reads: 0) barcode41 (Reads: 4) barcode42 (Reads: 4) barcode43 (Reads: 2)<br>barcode44 (Reads: 2) barcode45 (Reads: 0) barcode46 (Reads: 17) barcode47 (Reads: 8) barcode48 (Reads: 6)<br>barcode49 (Reads: 28) barcode50 (Reads: 1) barcode51 (Reads: 0) barcode52 (Reads: 5) barcode53 (Reads: 1) barcode54 (Reads: 1)<br>barcode55 (Reads: 3) barcode56 (Reads: 5) barcode57 (Reads: 2) barcode58 (Reads: 181) barcode59 (Reads: 0)<br>barcode60 (Reads: 1) barcode61 (Reads: 2) barcode62 (Reads: 1) barcode63 (Reads: 4) barcode64 (Reads: 6) barcode65 (Reads: 5)<br>barcode66 (Reads: 5) barcode67 (Reads: 1) barcode68 (Reads: 3) barcode69 (Reads: 2) barcode70 (Reads: 3) barcode71 (Reads: 4)<br>barcode72 (Reads: 1) barcode73 (Reads: 3) barcode74 (Reads: 0) barcode75 (Reads: 2) barcode76 (Reads: 1) barcode77 (Reads: 1)<br>barcode78 (Reads: 1) barcode79 (Reads: 1) barcode80 (Reads: 1) barcode81 (Reads: 0) barcode82 (Reads: 0) barcode83 (Reads: 2)<br>barcode84 (Reads: 1) barcode85 (Reads: 0) barcode86 (Reads: 1) barcode87 (Reads: 2) barcode88 (Reads: 1) barcode89 (Reads: 2)<br>barcode90 (Reads: 0) barcode91 (Reads: 3) barcode92 (Reads: 2) barcode93 (Reads: 4) barcode94 (Reads: 1) barcode95 (Reads: 1)<br>barcode96 (Reads: 1)

CUMULATIVE OUTPUT

The cumulative output shows the total amount of bases or reads sequenced over time by your device.

Bases

Legend

- **Estimated** — Predicted total number of bases, prior to basecalling
- **Passed** — Bases equal to or above the quality score threshold.
- **Failed** — Bases below the quality score threshold.

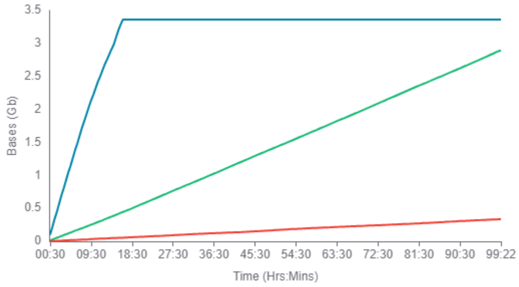

Reads

Legend

- **Total** — Total number of reads, including passed, failed and skipped
- **Passed** — Reads equal to or above the quality score threshold.
- **Failed** — Reads below the quality score threshold.
- **Skipped** — Reads that will not be basecalled. Post run basecalling is possible.

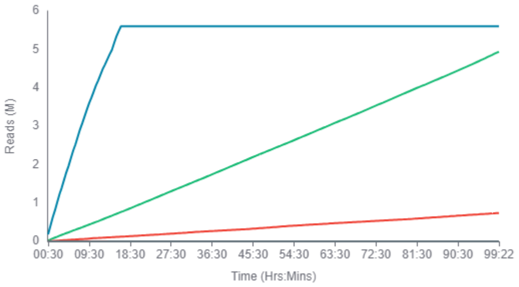

QUALITY SCORE

The quality score is calculated as basecalling is performed on your device. Reads that fall below the minimum value of 9 will be classified as failed reads. You can alter the accepted minimum quality score in MinKNOW.

Legend

- **Mode** — The most frequent quality score of reads in the run.
- **Spread** — The spread of quality scores, found by calculating full width half maximum.
- **Min. quality score** — Minimum quality score to be accepted as a passed read.

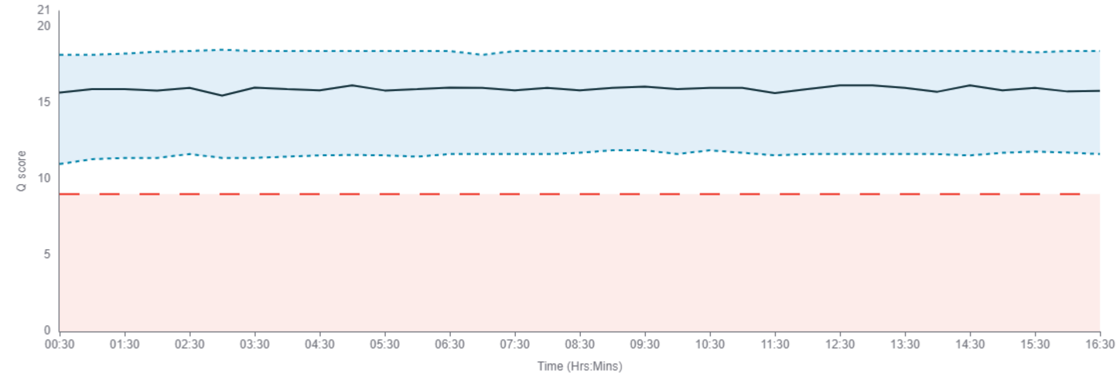

Troubleshooting

Quality score low

This can be due to the translocation speed being out of the accepted range, which can correlate to low quality scores. If you see that the translocation speed is out of the accepted range in the below graph, please see the Flow Cell refuelling page linked here for further troubleshooting.

Run health

PORE ACTIVITY

The Pore activity graph shows the performance of your sample as it is being sequenced during a run.

Legend

- **Sequencing** — Pore currently sequencing
- **Adapter** — Pore currently sequencing adapter
- **Pore available** — Pore available for sequencing
- **Unavailable** — Pore unavailable for sequencing
- **Active feedback** — Channel ejecting analyte
- **No pore** — No pore detected in channel
- **Out of range-high** — Current is positive but unavailable for sequencing
- **Out of range-low** — Current is negative but unavailable for sequencing
- **Multiple** — Multiple pores detected. Unavailable for sequencing.
- **Saturated** — The channel has switched off as current levels exceed hardware limitations
- **Zero** — Pore currently unavailable for sequencing
- **Channel disabled** — Channel is disabled and awaiting another pore scan
- **Unclassified** — Pore status unknown

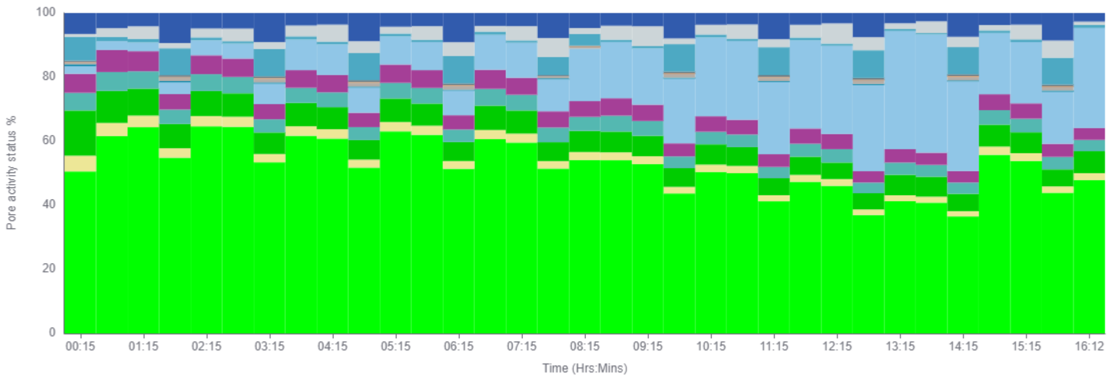

Troubleshooting

General

Some commonly seen issues are excess pores classified as Recovering, Open Pore, or Free Adapter. To find out what advice is applicable for your run, visit the user guide.

PORE SCAN

A Pore scan is performed at configurable time intervals to determine the current status of pores within channels on a Flow Cell. For this run a Pore scan is performed every 1.5 hrs.

Legend

- **Pore available** — Pore in channel available for sequencing
- **Reserved pore** — Pore in reserve, will return to available when required
- **Unavailable** — Pore inhibited from sequencing
- **Saturated** — Possible contamination in the sample
- **Zero** — No current is passing through this pore, possibly due to bubbles on the membrane
- **Inactive** — Pore no longer suitable for further sequencing

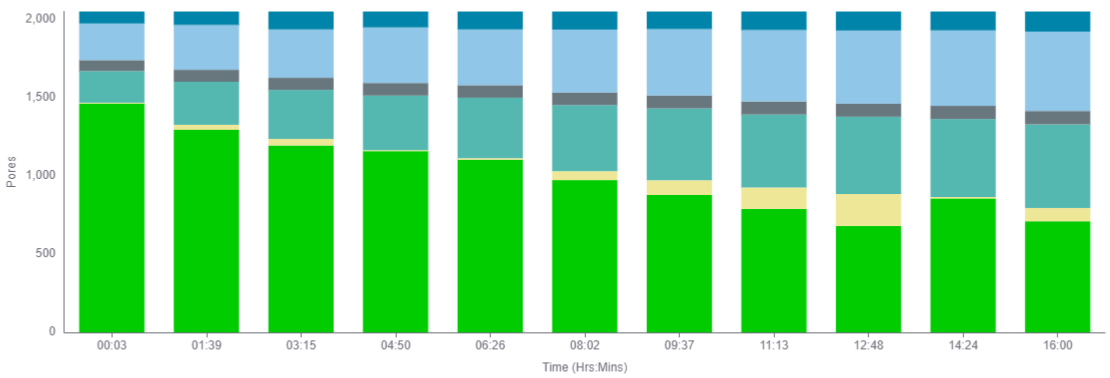

Troubleshooting

High proportion Unavailable
Possible contaminants in library blocking the pore. Consider using the Flow Cell Wash Kit, and reloading a library.

High proportion Inactive
If localised to one area of the Flow Cell, this could indicate that an air bubble has been introduced during the flushing/loading steps. If inactivity is spread across the Flow Cell this could be caused by improper loading of the library, please refer to the user guide for further support.

TRANSLOCATION SPEED

The translocation speed is the rate at which DNA/RNA travels through pores as it is being sequenced.

Legend

Median | 75% quartile | 25% quartile | Accepted range

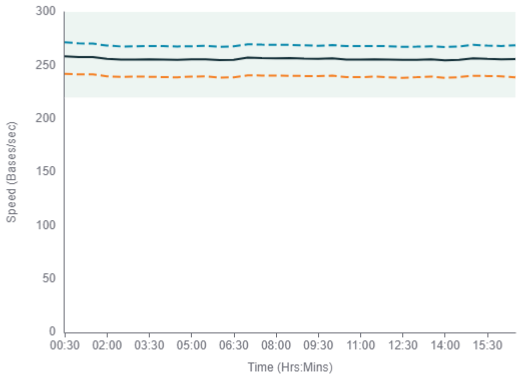

Troubleshooting

Low speed
Check that the Flow Cell is within the target temperature range.

Note
Low-quality and short reads are not included in this graph.

TEMPERATURE

The temperature of the Flow Cell over the run time.

Legend

Measured | Target

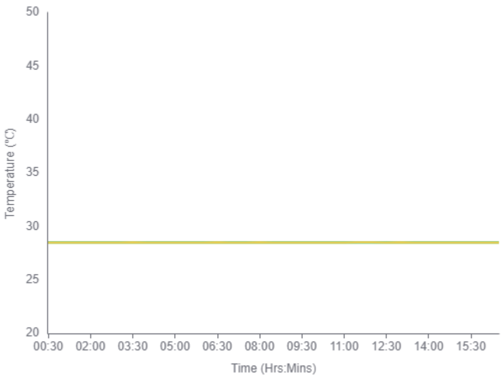

Troubleshooting

Out of range
Check that the Flow Cell is correctly seated and firmly pushed down into the device. Ensure ambient temperature is always within the specified range for your device in the user guide.

Air flow should be good but not excessive. Excessive amounts of cool air blowing on the device could prevent it from reaching target temperature.

Run log

SYSTEM MESSAGES

System messages are a record of the events that occurred in the time covered by this report.

Errors

None

Warnings

Disk usage alert · 07 Aug 23, 20:03
Disk usage alert – you only have 197 GB of space free, which is insufficient for the run. Please free up some space, otherwise your run will stop in approximately 1d 12h 10m.

Events

Disk space · 07 Aug 23, 08:16
Disk /data has 247 GB space remaining

Waiting for temperature · 07 Aug 23, 08:16
Waiting up to 300 seconds for temperature to stabilise at 28.5°C

Starting · 07 Aug 23, 08:17
Starting sequencing procedure

Pore scan starting · 07 Aug 23, 08:17
Performing Pore Scan

Pore scan result · 07 Aug 23, 08:21
Pore scan for flow cell FAW08908 has found a total of 1466 pores. 498 pores available for immediate sequencing

Pore scan starting · 07 Aug 23, 09:52
Performing Pore Scan

Pore scan result · 07 Aug 23, 09:56
Pore scan for flow cell FAW08908 has found a total of 1325 pores. 488 pores available for immediate sequencing

Pore scan starting · 07 Aug 23, 11:28
Performing Pore Scan

Pore scan result · 07 Aug 23, 11:32
Pore scan for flow cell FAW08908 has found a total of 1235 pores. 463 pores available for immediate sequencing

Pore scan starting · 07 Aug 23, 13:03
Performing Pore Scan

Pore scan result · 07 Aug 23, 13:08
Pore scan for flow cell FAW08908 has found a total of 1163 pores. 467 pores available for immediate sequencing

Pore scan starting · 07 Aug 23, 14:39
Performing Pore Scan

Pore scan result · 07 Aug 23, 14:43
Pore scan for flow cell FAW08908 has found a total of 1112 pores. 456 pores available for immediate sequencing

Pore scan starting · 07 Aug 23, 16:14
Performing Pore Scan

Pore scan result · 07 Aug 23, 16:19
Pore scan for flow cell FAW08908 has found a total of 1029 pores. 422 pores available for immediate sequencing

Pore scan starting · 07 Aug 23, 17:50
Performing Pore Scan

Pore scan result · 07 Aug 23, 17:54
Pore scan for flow cell FAW08908 has found a total of 971 pores. 386 pores available for immediate sequencing

Pore scan starting · 07 Aug 23, 19:26
Performing Pore Scan

Pore scan result · 07 Aug 23, 19:30
Pore scan for flow cell FAW08908 has found a total of 925 pores. 371 pores available for immediate sequencing

Pore scan starting · 07 Aug 23, 21:01
Performing Pore Scan

Pore scan result · 07 Aug 23, 21:06
Pore scan for flow cell FAW08908 has found a total of 883 pores. 323 pores available for immediate sequencing

Pore scan starting · 07 Aug 23, 22:37
Performing Pore Scan

Pore scan result · 07 Aug 23, 22:41
Pore scan for flow cell FAW08908 has found a total of 864 pores. 414 pores available for immediate sequencing

Pore scan starting · 08 Aug 23, 00:12
Performing Pore Scan

Pore scan result · 08 Aug 23, 00:17
Pore scan for flow cell FAW08908 has found a total of 794 pores. 352 pores available for immediate sequencing

Message · 08 Aug 23, 00:56
Flow cell disconnected

UNIT ABBREVIATIONS

Byte	B
Kilobyte	KB
Megabyte	MB
Gigabyte	GB
Terabyte	TB

Base	b
Kilobase	kb
Megabase	Mb
Gigabase	Gb
Terabase	Tb

Minutes	mins
Hours	hrs
