# Supplementary material for: Identification and molecular characterization of Mycobacterium bovis DNA in GeneXpert® MTB/RIF ultra-positive, culture-negative sputum from a rural community in South Africa
Source: One Health. 2024 Mar 3;18:100702. doi: 10.1016/j.onehlt.2024.100702 (PMC10937233; doi:10.1016/j.onehlt.2024.100702)
Supplement: Supplementary material 7 — European Nucleotide Archive specific project code and accession numbers for all sequences generated. [file mmc8.pdf]

49 Spadina Ave. Suite 200
Toronto ON M5V 2J1 Canada
www.biorender.com

Confirmation of Publication and Licensing Rights

January 17th, 2024
Science Suite Inc.

Subscription:	*Postdoc Plan*
Agreement number:	*VF26CK2DJ7*
Journal name:	*One Health*

To whom this may concern,

This document is to confirm that Danai Etter has been granted a license to use the BioRender content, including icons, templates and other original artwork, appearing in the attached completed graphic pursuant to BioRender's Academic License Terms. This license permits BioRender content to be sublicensed for use in journal publications.

All rights and ownership of BioRender content are reserved by BioRender. All completed graphics must be accompanied by the following citation: "Created with BioRender.com".

BioRender content included in the completed graphic is not licensed for any commercial uses beyond publication in a journal. For any commercial use of this figure, users may, if allowed, recreate it in BioRender under an Industry BioRender Plan.

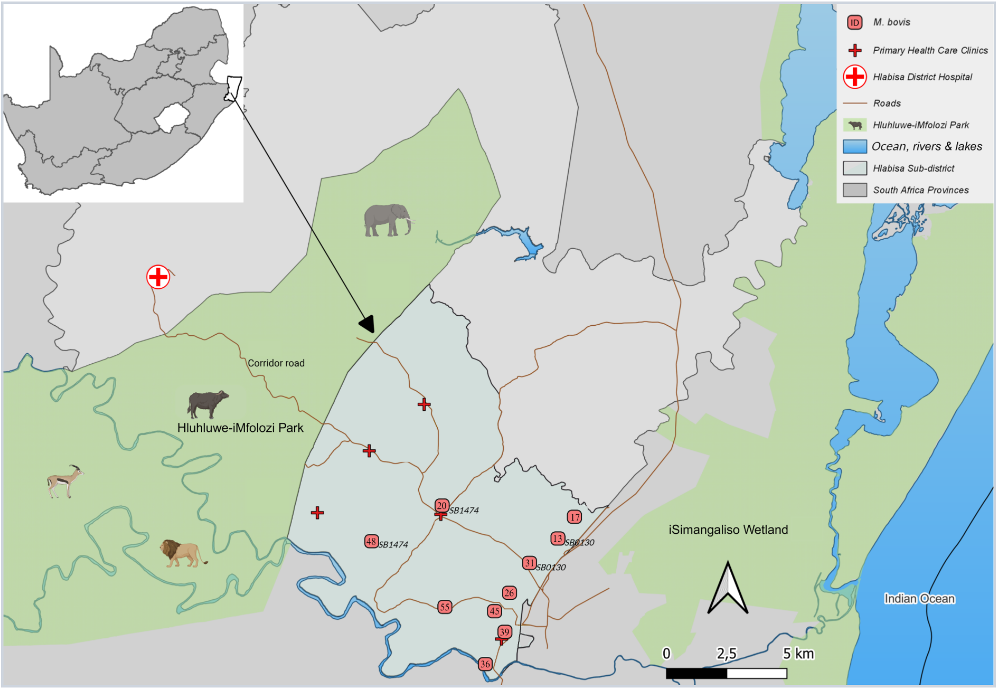

For any questions regarding this document, or other questions about publishing with BioRender refer to our BioRender Publication Guide, or contact BioRender Support at support@biorender.com.
