# Supplementary material for: Identification and molecular characterization of Mycobacterium bovis DNA in GeneXpert® MTB/RIF ultra-positive, culture-negative sputum from a rural community in South Africa
Source: One Health. 2024 Mar 3;18:100702. doi: 10.1016/j.onehlt.2024.100702 (PMC10937233; doi:10.1016/j.onehlt.2024.100702)
Supplement: Supplementary material 9 — Vukuzazi Team: Staff who significantly contributed to the implementation and conduct of Vukuzazi. [file mmc10.zip › report__20230807_0817_f8863b05.html]

MinKNOW Run Report-07-08-2023-FAW08908


# MinION Mk1C (MC-114017) Final report

07 Aug 23, 08:16 — 08 Aug 23, 00:42 · 20230807\_AHRI5sigsputumzTB · no\_sample · MC-114017

Protocol run ID: f8863b05-eca8-4225-ac76-2fc426a8f9f8

Run summary
Run configuration
Sequence output
Run health
Run log

Run summary

## DATA OUTPUT

Estimated bases

3.37 Gb

Data produced

90.43 GB

Reads generated

5.6 M

Estimated N50

873

## BASECALLING

Reads called

100%

Bases called (min Q score: 9)

2.9 Gb

335.32 Mb

Pass

Fail

## RUN DURATION

#### Elapsed time

80 hours of 80 hours

#### Run status

#### Stopped by user

View unit abbreviations used in this report

Run configuration

## RUN SETUP

Flow cell type

FLO-MIN114

Flow cell type alias

FLO-MIN114

Flow cell ID

FAW08908

Kit type

SQK-NBD114-96

## RUN SETTINGS

Specified run length

80 hrs

Active channel selection

On

Pore scan freq.

1.5 hrs

Reserved pores

On

Minimum read length

20 bp

Read splitting

On

Basecalling

High-accuracy model, 260 bps

Modified basecalling

Off

Trim barcodes

Off

Mid-read barcode filtering

Off

## DATA OUTPUT SETTINGS

FAST5 output

vbz\_compress

FAST5 reads per file

4000

FASTQ output

gzip\_compress

FASTQ reads per file

4000

BAM output

Off

Bulk file output

Off

Data location

/data/./20230807\_AHRI5sigsputumzTB/no\_sample/20230807\_0816\_MC-114017\_FAW08908\_f8863b05

## SOFTWARE VERSIONS

MinKNOW

22.12.5

Bream

7.4.8

Configuration

5.4.7

Guppy

6.4.6

MinKNOW Core

5.4.3

Sequence output

## READ LENGTHS · OUTLIERS REMOVED

The read length graph shows the total number of bases vs the read length. The longest 1% of strands are classified as outliers, and excluded to allow focus on the main body of data.

#### Legend

Basecalled

Estimated

Estimated N50

873

% Basecalled

100%

## OUTLIERS

The longest 1% of strands are classified as outliers, and aggregated into groups to show their relative amounts.

| Read length (kb) | Aggregated reads (Mb) |
| --- | --- |
| 0 - 128 | 28.31 |
| 128 - 256 | 0.15 |
| 256 - 384 | None |
| 384 - 492 | 0.5 |

## BARCODED READS

The total number of bases for each barcode is calculated and displayed below.

| Total bases (Gb) | Barcodes |
| --- | --- |
| 0–1 | barcode01 (Reads: 270312)  barcode02 (Reads: 279151)  barcode03 (Reads: 258272)  barcode04 (Reads: 299275)  barcode05 (Reads: 327746)  barcode06 (Reads: 313714)  barcode07 (Reads: 330402)  barcode08 (Reads: 320241)  barcode09 (Reads: 324697)  barcode10 (Reads: 337639)  barcode11 (Reads: 638903)  barcode12 (Reads: 302724)  barcode13 (Reads: 6537)  barcode14 (Reads: 1)  barcode15 (Reads: 4)  barcode16 (Reads: 1)  barcode17 (Reads: 2)  barcode18 (Reads: 7)  barcode19 (Reads: 0)  barcode20 (Reads: 1)  barcode21 (Reads: 3)  barcode22 (Reads: 8)  barcode23 (Reads: 0)  barcode24 (Reads: 4)  barcode25 (Reads: 1)  barcode26 (Reads: 2)  barcode27 (Reads: 2)  barcode28 (Reads: 2)  barcode29 (Reads: 3)  barcode30 (Reads: 0)  barcode31 (Reads: 1)  barcode32 (Reads: 6)  barcode33 (Reads: 4)  barcode34 (Reads: 4)  barcode35 (Reads: 1)  barcode36 (Reads: 0)  barcode37 (Reads: 0)  barcode38 (Reads: 0)  barcode39 (Reads: 2)  barcode40 (Reads: 0)  barcode41 (Reads: 4)  barcode42 (Reads: 4)  barcode43 (Reads: 2)  barcode44 (Reads: 2)  barcode45 (Reads: 0)  barcode46 (Reads: 17)  barcode47 (Reads: 8)  barcode48 (Reads: 6)  barcode49 (Reads: 28)  barcode50 (Reads: 1)  barcode51 (Reads: 0)  barcode52 (Reads: 5)  barcode53 (Reads: 1)  barcode54 (Reads: 1)  barcode55 (Reads: 3)  barcode56 (Reads: 5)  barcode57 (Reads: 2)  barcode58 (Reads: 181)  barcode59 (Reads: 0)  barcode60 (Reads: 1)  barcode61 (Reads: 2)  barcode62 (Reads: 1)  barcode63 (Reads: 4)  barcode64 (Reads: 6)  barcode65 (Reads: 5)  barcode66 (Reads: 5)  barcode67 (Reads: 1)  barcode68 (Reads: 3)  barcode69 (Reads: 2)  barcode70 (Reads: 3)  barcode71 (Reads: 4)  barcode72 (Reads: 1)  barcode73 (Reads: 3)  barcode74 (Reads: 0)  barcode75 (Reads: 2)  barcode76 (Reads: 1)  barcode77 (Reads: 1)  barcode78 (Reads: 1)  barcode79 (Reads: 1)  barcode80 (Reads: 1)  barcode81 (Reads: 0)  barcode82 (Reads: 0)  barcode83 (Reads: 2)  barcode84 (Reads: 1)  barcode85 (Reads: 0)  barcode86 (Reads: 1)  barcode87 (Reads: 2)  barcode88 (Reads: 1)  barcode89 (Reads: 2)  barcode90 (Reads: 0)  barcode91 (Reads: 3)  barcode92 (Reads: 2)  barcode93 (Reads: 4)  barcode94 (Reads: 1)  barcode95 (Reads: 1)  barcode96 (Reads: 1) |

## CUMULATIVE OUTPUT

The cumulative output shows the total amount of bases or reads sequenced over time by your device.

### Bases

#### Legend

Estimated

Predicted total number of bases, prior to basecalling

Passed

Bases equal to or above the quality score threshold.

Failed

Bases below the quality score threshold.

### Reads

#### Legend

Total

Total number of reads, including passed, failed and skipped.

Passed

Reads equal to or above the quality score threshold.

Failed

Reads below the quality score threshold.

Skipped

Reads that will not be basecalled. Post run basecalling is possible.

## QUALITY SCORE

The quality score is calculated as basecalling is performed on your device. Reads that fall below the minimum value of 9 will be classified as failed reads. You can alter the accepted minimum quality score in MinKNOW.

#### Legend

Mode

The most frequent quality score of reads in the run.

Spread

The spread of quality scores, found by calculating full width half maximum.

Min. quality score

Minimum quality score to be accepted as a passed read.


#### Troubleshooting

Quality score low

This can be due to the translocation speed being out of the accepted range, which can correlate to low quality scores. If you see that the translocation speed is out of the accepted range in the below graph, please see the Flow Cell refuelling page linked here for further troubleshooting.

Run health

## PORE ACTIVITY

The Pore activity graph shows the performance of your sample as it is being sequenced during a run.

#### Legend

Sequencing

Pore currently sequencing

Adapter

Pore currently sequencing adapter

Pore available

Pore available for sequencing

Unavailable

Pore unavailable for sequencing

Active feedback

Channel ejecting analyte

No pore

No pore detected in channel

Out of range-high

Current is positive but unavailable for sequencing

Out of range-low

Current is negative but unavailable for sequencing

Multiple

Multiple pores detected. Unavailable for sequencing.

Saturated

The channel has switched off as current levels exceed hardware limitations

Zero

Pore currently unavailable for sequencing

Channel disabled

Channel is disabled and awaiting another pore scan

Unclassified

Pore status unknown


#### Troubleshooting

General

Some commonly seen issues are excess pores classified as Recovering, Open Pore, or Free Adapter. To find out what advice is applicable for your run, visit the user guide.

## PORE SCAN

A Pore scan is performed at configurable time intervals to determine the current status of pores within channels on a Flow Cell. For this run a Pore scan is performed every 1.5 hrs.

#### Legend

Pore available

Pore in channel available for sequencing

Reserved pore

Pore in reserve, will return to available when required

Unavailable

Pore inhibited from sequencing

Saturated

Possible contamination in the sample

Zero

No current is passing through this pore, possibly due to bubbles on the membrane

Inactive

Pore no longer suitable for further sequencing


#### Troubleshooting

High proportion Unavailable

Possible contaminants in library blocking the pore. Consider using the Flow Cell Wash Kit, and reloading a library.

High proportion Inactive

If localised to one area of the Flow Cell, this could indicate that an air bubble has been introduced during the flushing/loading steps. If inactivity is spread across the Flow Cell this could be caused by improper loading of the library, please refer to the user guide for further support.

## TRANSLOCATION SPEED

The translocation speed is the rate at which DNA/RNA travels through pores as it is being sequenced.

#### Legend

Median

75% quartile

25% quartile

Accepted range


#### Troubleshooting

Low speed

Check that the Flow Cell is within the target temperature range.

Note

Low-quality and short reads are not included in this graph.

## TEMPERATURE

The temperature of the Flow Cell over the run time.

#### Legend

Measured

Target


#### Troubleshooting

Out of range

Check that the Flow Cell is correctly seated and firmly pushed down into the device. Ensure ambient temperature is always within the specified range for your device in the user guide.

Air flow should be good but not excessive. Excessive amounts of cool air blowing on the device could prevent it from reaching target temperature.

Run log

## SYSTEM MESSAGES

System messages are a record of the events that occurred in the time covered by this report.

Errors

None

Warnings

Disk usage alert
·
07 Aug 23, 20:03

Disk usage alert – you only have 197 GB of space free, which is insufficient for the run. Please free up some space, otherwise your run will stop in approximately 1d 12h 10m.

Events

Disk space
·
07 Aug 23, 08:16

Disk /data has 247 GB space remaining

Waiting for temperature
·
07 Aug 23, 08:16

Waiting up to 300 seconds for temperature to stabilise at 28.5°C

Starting
·
07 Aug 23, 08:17

Starting sequencing procedure

Pore scan starting
·
07 Aug 23, 08:17

Performing Pore Scan

Pore scan result
·
07 Aug 23, 08:21

Pore scan for flow cell FAW08908 has found a total of 1466 pores. 498 pores available for immediate sequencing

Pore scan starting
·
07 Aug 23, 09:52

Performing Pore Scan

Pore scan result
·
07 Aug 23, 09:56

Pore scan for flow cell FAW08908 has found a total of 1325 pores. 488 pores available for immediate sequencing

Pore scan starting
·
07 Aug 23, 11:28

Performing Pore Scan

Pore scan result
·
07 Aug 23, 11:32

Pore scan for flow cell FAW08908 has found a total of 1235 pores. 463 pores available for immediate sequencing

Pore scan starting
·
07 Aug 23, 13:03

Performing Pore Scan

Pore scan result
·
07 Aug 23, 13:08

Pore scan for flow cell FAW08908 has found a total of 1163 pores. 467 pores available for immediate sequencing

Pore scan starting
·
07 Aug 23, 14:39

Performing Pore Scan

Pore scan result
·
07 Aug 23, 14:43

Pore scan for flow cell FAW08908 has found a total of 1112 pores. 456 pores available for immediate sequencing

Pore scan starting
·
07 Aug 23, 16:14

Performing Pore Scan

Pore scan result
·
07 Aug 23, 16:19

Pore scan for flow cell FAW08908 has found a total of 1029 pores. 422 pores available for immediate sequencing

Pore scan starting
·
07 Aug 23, 17:50

Performing Pore Scan

Pore scan result
·
07 Aug 23, 17:54

Pore scan for flow cell FAW08908 has found a total of 971 pores. 386 pores available for immediate sequencing

Pore scan starting
·
07 Aug 23, 19:26

Performing Pore Scan

Pore scan result
·
07 Aug 23, 19:30

Pore scan for flow cell FAW08908 has found a total of 925 pores. 371 pores available for immediate sequencing

Pore scan starting
·
07 Aug 23, 21:01

Performing Pore Scan

Pore scan result
·
07 Aug 23, 21:06

Pore scan for flow cell FAW08908 has found a total of 883 pores. 323 pores available for immediate sequencing

Pore scan starting
·
07 Aug 23, 22:37

Performing Pore Scan

Pore scan result
·
07 Aug 23, 22:41

Pore scan for flow cell FAW08908 has found a total of 864 pores. 414 pores available for immediate sequencing

Pore scan starting
·
08 Aug 23, 00:12

Performing Pore Scan

Pore scan result
·
08 Aug 23, 00:17

Pore scan for flow cell FAW08908 has found a total of 794 pores. 352 pores available for immediate sequencing

Message
·
08 Aug 23, 00:56

Flow cell disconnected

UNIT ABBREVIATIONS

Byte

B

Kilobyte

KB

Megabyte

MB

Gigabyte

GB

Terabyte

TB

Base

b

Kilobase

kb

Megabase

Mb

Gigabase

Gb

Terabase

Tb

Minutes

mins

Hours

hrs
